# *In Vitro* Site Selection of a Consensus Binding Site for the *Drosophila melanogaster* Tbx20 Homolog Midline

**DOI:** 10.1371/journal.pone.0048176

**Published:** 2012-10-25

**Authors:** Nima Najand, Jae-Ryeon Ryu, William J. Brook

**Affiliations:** Genes and Development Research Group, Alberta Children’s Hospital Research Institute, Department of Biochemistry and Molecular Biology, University of Calgary, Calgary, Alberta, Canada; Karlsruhe Institute of Technology, Germany

## Abstract

We employed *in vitro* site selection to identify a consensus binding sequence for the *Drosophila melanogaster* Tbx20 T-box transcription factor homolog Midline. We purified a bacterially expressed T-box DNA binding domain of Midline, and used it in four rounds of precipitation and polymerase-chain-reaction based amplification. We cloned and sequenced 54 random oligonucleotides selected by Midline. Electromobility shift-assays confirmed that 27 of these could bind the Midline T-box. Sequence alignment of these 27 clones suggests that Midline binds as a monomer to a consensus sequence that contains an AGGTGT core. Thus, the Midline consensus binding site we define in this study is similar to that defined for vertebrate Tbx20, but differs from a previously reported Midline binding sequence derived through site selection.

## Introduction

The T-box family of transcription factors plays numerous developmental roles in metazoans [1]. Recent evidence shows that T-box genes are an ancient family of transcription factors that predate the appearance of the Metazoa [2]. The unifying domain in this gene family is a highly conserved ∼180 amino acid DNA binding domain, called the T-box, named after the founding member Brachyury (T). The Mouse T gene was also the first T-box transcription factor for which the DNA binding motif was identified [3]. The motif consists of a 24 base-pair (bp) palindrome which has come to be known as the T-site (AATTTCACACCT-AGGTGTGAAATT). Since then, several reports have shown that other T-box family members have some affinity for the full T-site, or the T half-site which consists of only half of the palindrome [4]. Site selection experiments have also been performed on Tbx5 [5], [6], Tbx6 [7], Xbra [8], Eomsodermin [8], VegT [8], Spt [9], Ntl [9], and Tbx20 [6]. Every one of these T-box proteins has a strong preference for oligonucleotides that contain a GGTGT core with some variability in the nucleotides flanking this core.

**Figure 1:**
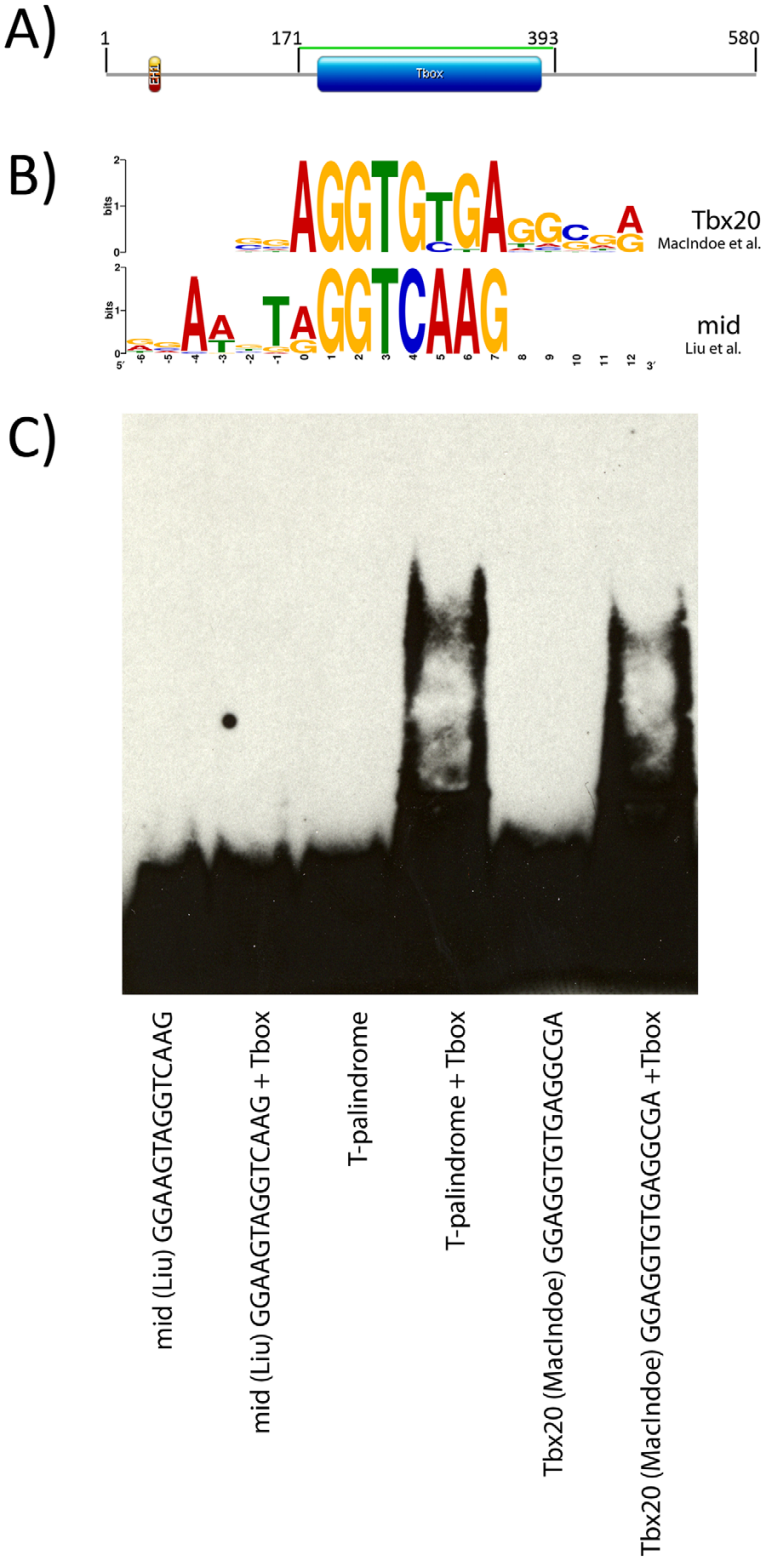
Comparison of previously identified motifs of midline and Tbx20. **A)** A schematic of *D. melanogaster* Midline protein based on clone RE27439 drawn using Prosite MyDomains [42]. The fragment used in our analysis – green line (amino acids 171–393) spans the DNA binding T-box domain – blue box (amino acids 187–383). The EH1domain [19] in the N-terminal region is in orange. **B)** The DNA binding motif of mouse Tbx20 is derived from the site selection data presented by Macindoe et al. [6], while the mid DNA binding motif was generated from data by Liu et al. [18]. Comparison of the aligned motifs show that the two homologues only have positions 0–3 in common. Nucleotides at all other positions differ, suggesting that Drosophila Mid recognizes a different consensus sequence than that bound by other Tbx20 proteins. **C)** The binding consensus identified by Liu et al. (GGAAGTAGGTCAAG ) [18], full Brachyury palindrome (T-palindrome AATTTCACACCTAGGTGTGAAATT) [3] and the Tbx20 consensus derived by MacIndoe et al. (GGAGGTGTGAGGCGA) [6] were tested on an EMSA for interaction with the T-box domain of bacterially expressed Mid.

In *Drosophila melanogaster* Midline (Mid) (Tbx20 in vertebrates) is involved in several aspects of development including segmentation, cardiogenesis, neurogenesis, and limb formation [10], [11], [12], [13], [14], [15], [16], [17]. However, the mechanisms by which Mid regulates these developmental processes is not well understood. To date, only four direct targets of Mid have been identified. These include components of the axon guidance pathway: Frazzled, Slit, and Robo [18]; and the segment polarity gene Wingless [19]. The direct regulation of Frazzled, Slit and Robo by Mid was discovered through identification of the Mid binding motif using site selection [18]. In that study, Liu et al. determined the Mid binding motif by incubating oligonucleotides with crude embryonic nuclear lysates and used an anti-Mid antibody to co-precipitate native Mid protein and the bound oligonucleotides. This experiment suggested that Mid selectively binds a 5′ GGAAGT**AGGT**CAAG consensus sequence (Figure 1B). The AGGT at positions 7–10 of this sequence (in bold) resembles the core AGGT found in the classic T-site. However, outside of this similarity many of the nucleotides within the core or flanking nucleotides do not match the T-site or other site selected T-box motifs, including the motif of the vertebrate homologue Tbx20 (Figure 1B). Strikingly, the nucleotide sequence GGTCAAG was present in 100% of the oligonucleotides selected by Mid, suggesting that there is an absolute requirement for the binding motif. However, no other T-box factors have displayed either a requirement or a preference for this sequence.

Through a site-selection experiment with bacterially expressed Mid T-box domain (Figure 1A) we identify a sequence similar to typical T-half sites but different from the site reported by Liu et al.

## Results and Discussion

### Mid T-box Domain does not Bind a GGTCAAG Motif in vitro

To investigate whether Mid is able to bind the novel T-box motif *in vitro* [18], we performed electro-mobility shift assays (EMSAs) using 5′ biotin-labeled oligonucleotides incubated with bacterially expressed, purified, C-terminal 6xHis-tagged Mid T-box domain (Mid_Tbx_) (Figure 1A). We used a 196 amino acid fragment of the full length Mid protein which contains the T-box domain because we were unable to express soluble, full length Mid. Research on other T-box transcription factors such as Tbx20 has been able to generate bonafide binding motifs using the DNA binding domain [6]. We found that Mid_Tbx_ was able to bind and retard the migration of oligonucleotides containing either the Brachyury T-site or the vertebrate Tbx20 site. However, when Mid_Tbx_ was incubated with the motif identified by Liu et al. (Figure 1B) we were unable to detect the presence of a lower mobility band (Figure 1C). This demonstrates that our bacterially expressed protein is capable of binding to DNA *in vitro* and that Mid_Tbx_ has an affinity for the full T-site and the Tbx20 site but is unable to bind to the motif identified by Liu et al.

### The Mid_Tbx_ Binding Motif Resembles a Classic T-half-site

**Figure 2:**
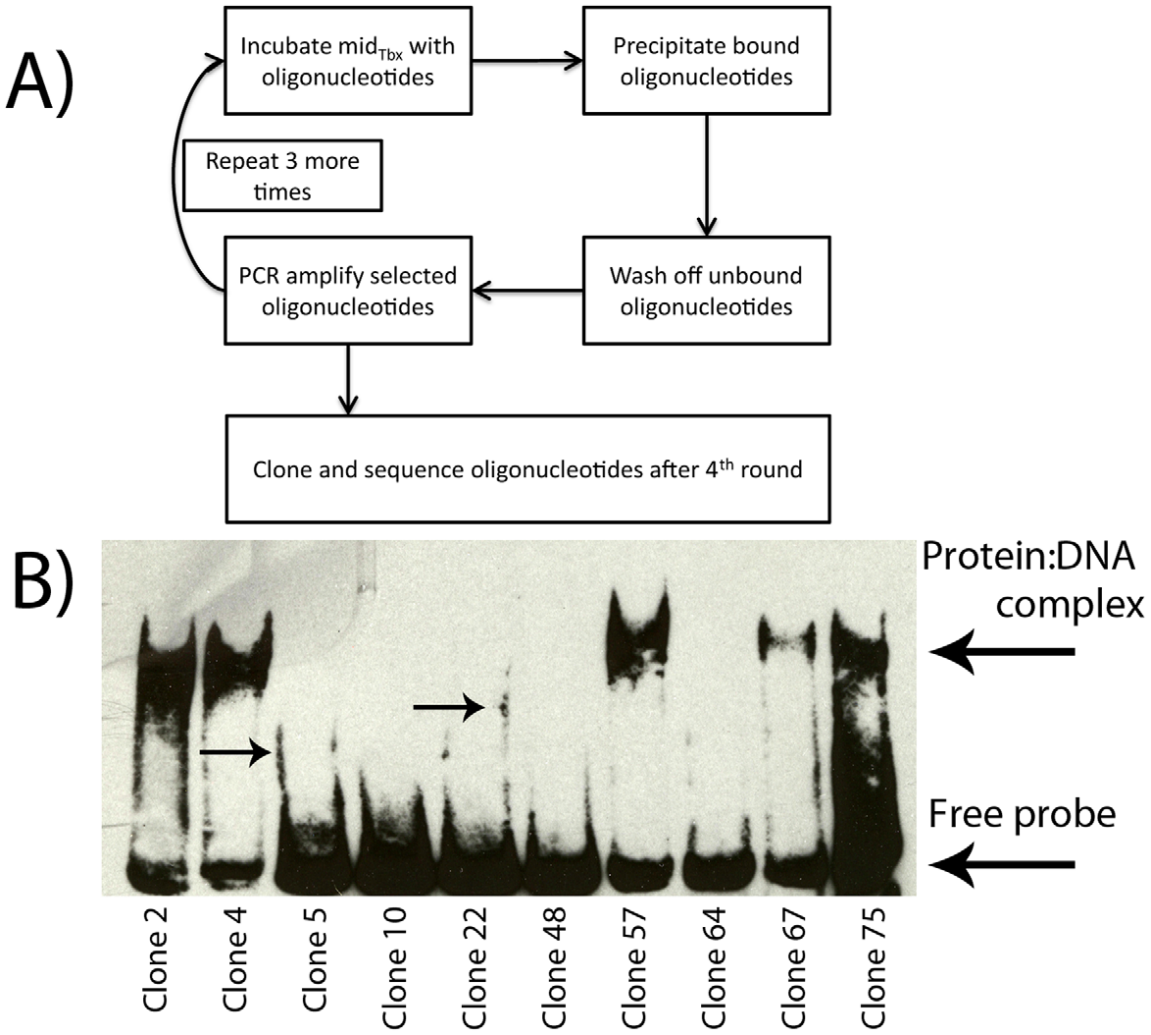
Site selection workflow and EMSAs on cloned fragments. **A)** Outline of the selection procedure carried out to determine the DNA binding motif of Mid. In the first round, oligonucleotides consisting of a random 26 nucleotide core flanked by primer sequences were incubated with mid_Tbx._ After purification and PCR amplification of co-precipitated fragments, the PCR amplified fragments were used in subsequent rounds of selection. **B)** All 54 oligonucleotide fragments were cloned and used as a template to create probes for EMSAs. Each probe was tested for recognition by Mid_Tbx._ Probes positive for shifts, such as 2, 4, 57, 67 and 57 were tested a minimum of two times. Probes negative for shifts, such as 5, 10, 22, 48, and 64 were tested 3–5 times to exclude the possibility of false negatives. Arrows point to streaks often seen in probes that were weakly bound. Probes that did not display a noticeable, shifted band were considered to be unrecognized.

In order to determine the preferred sequences bound by Mid, we performed a site selection experiment. Using buffer conditions nearly identical to those described by Liu et al., we incubated double-stranded oligonucleotides containing a random 26 bp core flanked by 25 bp primer sequences with purified Mid_Tbx_. Following precipitation of the nucleo-protein complex using nickel beads and magnets, we washed off the unbound oligonucleotides, eluted the Mid_Tbx_-DNA complexes, and PCR amplified the selected fragments through 4 rounds of selection. We then cloned and sequenced 54 different oligonucleotides (Figure 2A). To reduce the background of oligonucleotides precipitated due to weak or non-specific binding, each cloned oligonucelotide was used to generate a biotin-labelled probe and tested by EMSA. Probes were considered unshifted if they failed to produce a visible band at least once in a minimum of three independent EMSAs (Figure 2B). Mid_Tbx_ was able to shift 27 of the 54 cloned fragments in an EMSA (Figure 2B). Most (24/27) of the remaining probes displayed some evidence for binding to Mid_Tbx_ such as the appearance of streaks along the edges of the gel lanes (Figure 2B arrows). However, this transient or weak binding was considered insufficient to include the sequence of those probes in our analysis.

**Figure 3:**
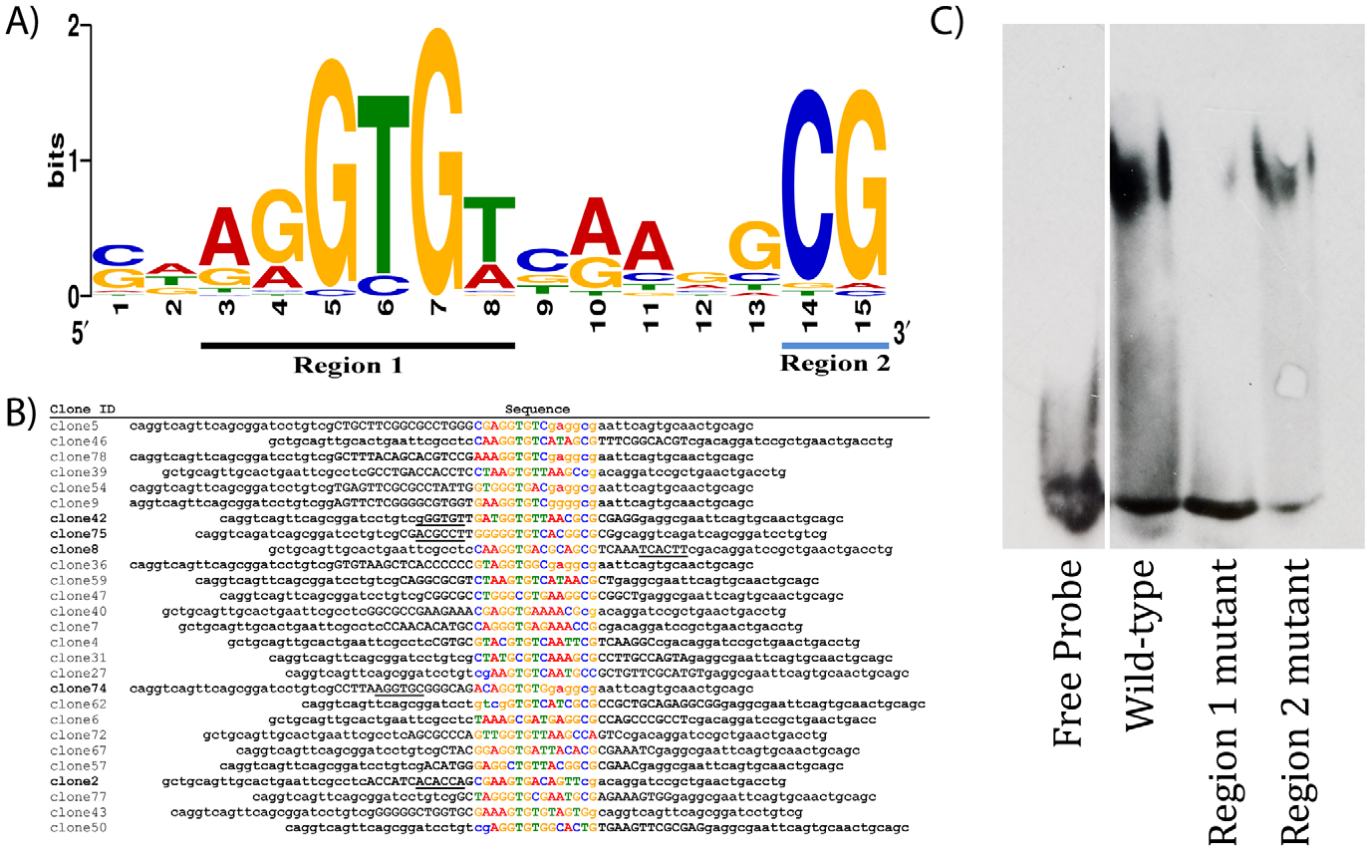
DNA motif selected by Mid_Tbx_. **A)** The sequence logo corresponding to the oligonucleotide selected by Mid_Tbx_ after 4 rounds of selection. The 27 EMSA verified sequences and the flanking primer sequences for some were input into MEME. MEME was set to use each nucleotide once and to generate a motif with a maximum length of 26 nucleotides. Region 1 and 2 are underlined in black and blue respectively. **B)** Aligned sequences of the 27 oligonucleotides used to generate the motif in A. The 15 nucleotides present in A are colour coded according to the nucleotide. Flanking sequences are in black. Nucleotides within the random 26 bp core are in uppercase, while those found within the primer sequences are in lowercase. The second potential Mid_Tbx_ binding site in oligonucleotides represented by clones 2, 8, 42, 74, and 75, has been underlined. **C)** An EMSA using the 15 base pair consensus motif identified in this study. The migration of oligonucleotides with wild-type Mid binding motif (CAAGGTGTCAAGGCG) is slowed in the presence of Mid_Tbx_. However, Mid_Tbx_ does not appear to have an affinity for oligonucleotides where region 1 has been mutated (CACCCCCCCAAGGCG). Oligonucleotides mutant for region 2 (CAAGGTGTCAAGGAA) are still bound and retarded by Mid_Tbx._

The corresponding sequences of the shifted probes were used to generate a binding motif using MEME software [20] and verified using SCOPE [21] and MochiView [22] (data not shown). Since all the probes were positive for an interaction with Mid_Tbx_, the parameters were set such that all 27 sequences were used to generate a motif. Our results show that Mid_Tbx_ selects a 15 bp motif corresponding to the sequence [CG][ATG][AG][GA]GTG[TA][CGT][AG]A[GA]GCG or SDRRGTGWBRARGCG (Figure 3A). A similar analysis of the remaining sequences that do not shift Mid_T-box_ could not generate a binding motif using MEME (data not shown). However, manual inspection of the sequences showed that most had sequences that resemble consensus T-box sites, suggesting that they may have been included in the selected oligonucleotides because of weak affinity for Mid_Tbx_.

The most frequent nucleotide at each position results in CAAGGTGTCAAGGCG as a consensus Mid_Tbx_ binding motif. This motif is comprised of two regions where Mid_Tbx_ displays a strong preference for particular nucleotides. Region 1 (Figure 3A – black underline) spans positions 3–8 and consists of an AGGTGT sequence identical to the T-half-site. Region 2 (Figure 3A – blue underline) consists of a CG at positions 14 and 15. The two regions are separated by 5 bases where there is a less strict requirement for particular nucleotides.

The nucleotides in region 1 (3–8) resemble the T-half-site that Mid_Tbx_ is able to bind specifically (Figure 1C). Furthermore, region 1 is similar to sites selected by other T-box family members [5], [6], [7], [8], [9]. In region 1, Mid_Tbx_ has a strong requirement for a GTG at positions 5–7 with the T at position 6 occasionally being substituted for a C. This substitution is not correlated to the presence of another nucleotide at other positions within the motif. Positions 3 and 4 appear to be more variable, most commonly consisting of purines while position 8 is often a T or A. This demonstrates that Mid_Tbx_ binds to a motif recognized by other members of the T-box family.

Region 2, consisting of CG at positions 14 and 15 has not yet been found in the binding motif of other T-box genes. We considered whether the presence of the CG was an artifact since it often appears in the primer sequence included in our analysis (Figure 3B). Two lines of evidence demonstrate that this is likely not the case. First, analysis of the same 27 clones without the primer sequences using MEME still produces a motif with a CG at positions 14 and 15 (not shown). This demonstrates that Mid_Tbx_ selects a CG dinucleotide at positions 14 and 15 within the random 26 nucleotide core or primer sequence. Second, the number of nucleotides between region 2 and region 1 is invariant between the clones. If region 2 was not specifically selected by Mid_Tbx,_ we would expect region 1 to vary in its location with respect to the primer sequence. However, since the spacing between region 1 and 2 is always exactly 5 nucleotides, we argue that there is a real preference for a CG dinucleotide at positions 14 and 15. However, there is not an absolute requirement for a CG in region 2 since Mid_Tbx_ can bind to clones 43, 50 and 72 which lack it (Figure 3). In addition, Mid_Tbx_ can bind the T-site and the Tbx20 motif, which both lack a CG (Figure 1C). The mouse Tbx20 site does contain a CG dinucleotide (GGAGGTGTGAGG**CG**A), but it is not as frequently represented as the CG in the Mid consensus and it is shifted over by one position such that it corresponds to nucleotides 13 and 14 numbered with respect to the Mid motif. Furthermore, the CG in the mouse Tbx20 motif is likely an artifact since it falls in the primer region.

In order to test whether region 2 is necessary for binding, we generated a 15 base oligonucleotide corresponding to our consensus (CAAGGTGTCAAGGCG ). The bases in region 1 or region 2 (underlined) were mutated in order to assess their effect on binding (Figure 3C). We found that mutating region 1 disrupted binding of Mid_Tbx_ on an EMSA, suggesting that region 1 is necessary and region 2 is not sufficient. Mutating the CG of region 2 did not appear to affect binding on an EMSA, suggesting that region 1 is necessary and region 2 is not.

There is no strong requirement for specific nucleotides between regions 1 and 2, as most nucleotides are represented at each position. Three of the four possible nucleotides are permitted while one is excluded at each position except at position 10, where Mid_Tbx_ seems to favour a purine.

### Mid_Tbx_ Binds as a Monomer to the Identified Motif

Previous studies on members of the T-box transcription factor family have shown that T-box genes such as human Tbx2 [23], Tbx3 [24], [25], Tbx5 [5], and mouse T [3], [26], T-bet [27], Tbx2 [28], Tbx6 [7] and Tbx20 [6] bind as monomers. Examples of T-box factors that bind DNA as dimers include human Tbx1 [23], Tbx6 [29] and Xbra [8], [23], [30]. Our data suggests that Mid_Tbx_ binds DNA as a monomer. On EMSAs we only observe one band when Mid is bound to the palindromic T-site which consists of two potential binding sites (Figure 1C). Transcription factors that bind DNA as a homodimer often display two bands on an EMSA - a higher mobility band belonging to a single protein bound to the DNA, and a lower mobility band representing a homodimer bound DNA. Some examples of T-box proteins displaying multiple EMSA bands on oligonucleotides with more than one binding site include T, Tbx5 and Tbx6 [5], [29], [31]. It is unlikely that the single band present in our study is due to an inability of Mid_Tbx_ to bind DNA as a monomer, since the single band appears at the same mobility as Mid_Tbx_ bound to the Tbx20 motif which contains only a single potential T-site (Figure 1C). Furthermore, we observe that Mid_Tbx_ is able to bind a Tbx20 motif that consists of only a “half-site” suggesting that a “full-site” containing two potential binding motifs is not necessary and thus Mid_Tbx_ is not an obligate dimer. Finally, 22 out of 27 oligonucleotides selected in our study only contain one apparent T-site while five others (clones 2, 8, 42, 74 and 75) have two sites present in different orientations and spacing (Figure 3B). This suggests that each site was selected by a Mid_Tbx_ monomer rather than a dimer, which would impose strict requirements on orientation and spacing. The five oligonucleotides with more than one potential binding site show only a single band on EMSAs. Because this band has the same mobility as oligonucleotides with only a single binding site, it suggests that either the two Mid_Tbx_ monomers bound to a single oligonucleotide are not sufficiently stable to resolve on a gel, or that one monomer sterically hinders the binding of another, or that there is enough excess probe that the proteins always bind a unique probe.

**Figure 4:**
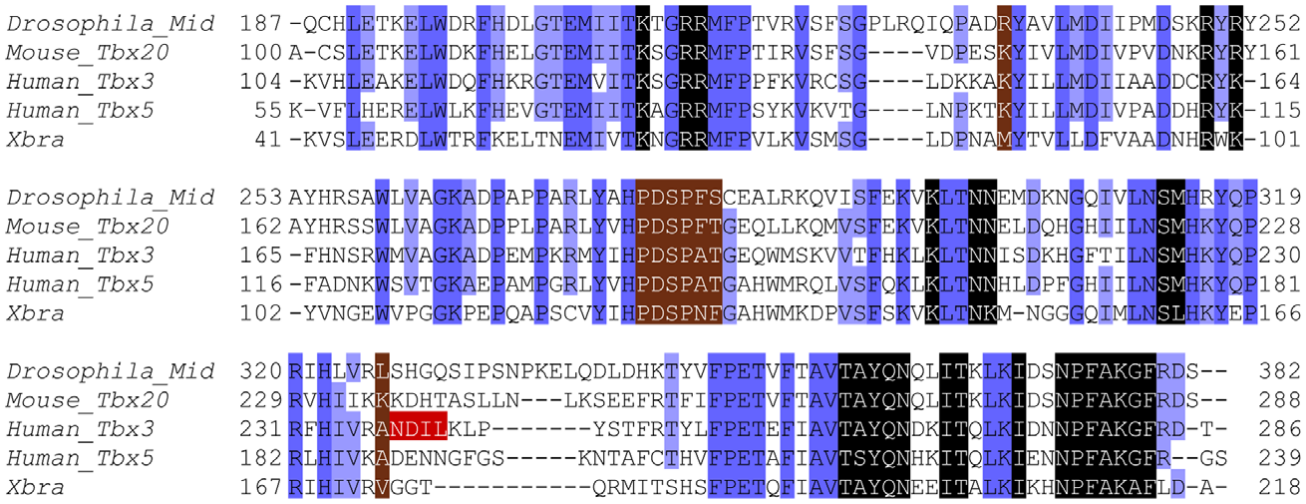
Protein sequence alignment of the T-box domain of select T-box genes. The T-box domain of Mid is aligned with its vertebrate homologue Tbx20 as well as T-box genes for which the crystal structure has been solved (obtained from Pfam and modified to remove gaps [43]). Amino acid residues conserved in all 5 members are in dark blue, while those found in 4 out of 5 are in a lighter shade of blue. Residues implicated in direct interactions with the DNA based on the crystal structures of Tbx3, Tbx5 and Xbra are highlighted in black [25], [30], [32]. Those that are involved in dimerization or monomer-monomer contacts in the Xbra cystals are highlighted in brown [25], [30]. Amino acids involved in the small monomer interface of Tbx3 are highlighted in red.

The sequences necessary for dimerization in other T-box factors are not conserved in Mid, which is also consistent with Mid binding as a monomer (Figure 4). Xbra homodimerizes through a relatively small interface of 250 Å^2^ found near the centre of the T-box domain [30]. The small polar N129 residue in Xbra is replaced with a large hydrophobic F281 in Mid and F130 in Xbra is replaced by S282 in Mid. Likewise Xbra M85 is substituted with R235, and Xbra V173 corresponds to L326 in Mid. Overall, 4 of the 8 dimerization residues are not conserved in *Drosophila* Mid. Furthermore, Tbx20 also differs from both Mid and Xbra at these same 4 positions (Figure 4). The crystal structure of Tbx3 bound to a palindromic T-site shows that the two monomers are rotated with respect to one another on the DNA strand and use different residues (238–241 on Tbx3) to contact one another [25]. These residues fall within a poorly conserved region of the T-box domain. Comparison to the corresponding Mid residues (327–330) shows that none of these amino acids are conserved (Figure 4). Similarly, only Tbx3 D239 is identical to the corresponding Tbx20 residue. The small monomer-monomer contacts defined in the Tbx3 crystal structure are thought to be insufficient to facilitate dimerization and as such, Tbx3 is believed to bind as a monomer [25]. Finally, the crystal structure of Tbx5 bound to a half-site shows that the regions responsible for monomer-monomer contacts in Tbx3 have low electron density suggesting that these domains are conformationally flexible and thus are unlikely to be in involved in dimerization [32].

Taken together, the site selection data and the comparison of the Mid amino acid sequence with evidence from the crystal structures of the Xbra, Tbx3 and Tbx5 suggest that Mid binds DNA as a monomer. We have also found that Mid is able to directly regulate the transcription of the *wingless* gene, *in vivo,* by binding to sequences within the *wg* enhancer [19]. The sequences Mid binds in order to regulate *wg* resemble the motif we present in this study (Figure 3). These *in vivo* Mid binding sites provide additional evidence that Mid is acting as a monomer.

### Discrepancy with Previously Reported Mid Binding Motif

The motif we identified does not contain the AGGTCAAG sequence identified by Liu et al. [18]. Furthermore, the AGGTCAAG motif was not detected in any of the oligonucleotides recovered in our site selection (Figure 3), nor was our purified protein able to shift the Liu et al. sequence on an EMSA (Figure 1C). The striking difference between the two motifs could arise for a number of reasons. First, in our study we used a bacterially expressed, C-terminally 6xHis-tagged Mid T-box domain (Figure 1A) whereas the previous motif was identified using a full-length protein purified from *Drosophila* nuclear lysates. It is possible that the full-length protein has different binding properties compared to the T-box domain. However, our motif resembles those from other studies which have used either full-length or the T-box domain of T-box genes to generate a binding motif [3], [5], [7], [8], [9], [33], [34]. This suggests that using the Mid DNA-binding domain should produce a valid binding motif.

Purification of native protein from nuclear lysates has the additional caveats that the purified protein may be post-translationally modified and that additional co-factors may be co-purified. While little is known about their post-translational modification, T-box factors have been shown to bind a variety of transcriptional co-factors. For example, Mid can bind the cardiac transcription factors Tinman and Pannier [35] while Tbx20 can bind the vertebrate homologues Nkx2.5 and Gata4 [33]. Mid and mouse Tbx15 and Tbx18 (closely related to Tbx20) bind the Groucho/Tle co-repressor [19], [34] and Mouse Tbx20, Tbx5 and Xbra have been shown to bind Smads [36], [37]. Tpit can bind the homeodomain protein Pitx [38] and VegT can physically interact with Tcf3 [39]. However, it is not known whether these factors influence the preferred T-box binding site. Furthermore, the predicted binding site for mouse Tbx20 generated from a genome-wide ChIP-seq experiment is very similar to other T-box consensus sequences including our own [40]. This makes it seem less likely that the differences between our study and that of Liu et al. are simply due to the source of the protein.

Finally, it is possible that non-specific binding of the antibody to other proteins within the lysate may in fact produce a motif for a different protein than that being studied. This possibility may explain the discrepancy between the Liu et al. motif and all other T-box transcription factors including the motif identified for Mid in the present study.

### Conclusions

T-box transcription factors have been shown to bind variations of the 24 bp palindromic Brachyury DNA binding motif called the T-site. It has been suggested that the specificity of T-box proteins for particular binding sites arises from the spacing and orientation of the two half-sites as well as the nucleotides flanking the core AGGTGT of each half-site [8]. We employed a site selection technique and identified DRRGTGWBRARGCG as the DNA binding motif for the Drosophila melanogaster Mid protein (Figure 3). The CG found at positions 14 and 15 in this motif appear to be specifically selected by Mid_Tbx_ but are not essential for binding in an EMSA (Figure 3C). The motif identified in Figure 3 resembles that of most other T-box transcription factors and in particular is very close to the motif identified for the vertebrate homologue of Mid, Tbx20 [6]. It does not, however, resemble the motif previously identified for Mid (Figure 1B) [18]. Furthermore, we find that Mid_Tbx_ is unable to shift the sequence identified by Liu et al. in an EMSA (Figure 1C).

Based on our results and analysis we propose that Mid binds DNA targets as a monomer. Five lines of evidence support this hypothesis: 1) Most oligonucleotides had a single site and when two half-sites were found (4/27 oligonucleotides) they were oriented and spaced randomly with respect to one another; 2) Mid_Tbx_ is able to bind oligonucleotides containing only a single binding site; 3) EMSAs using oligonucleotides containing two potential binding sites only display a single band that runs at approximately the same mobility as Mid_Tbx_ bound to a half-site; 4) The residues required for dimerization of Xbra and the non-stabilizing monomer-monomer contacts of Tbx3 are not conserved in Mid; 5) *in vivo* binding sites responsive to Mid are half-sites [19]. The possibility that region 2 in our motif is a variant half-site bound by a second Mid_Tbx_ monomer cannot be excluded and therefore a crystal structure of Mid_Tbx_ bound to this motif would be necessary to definitively conclude the nature of the Mid_Tbx_-DNA complex.

## Materials and Methods

### Expression of Mid T-box Domain

*Drosophila melanogaster* Midline T-box domain (residues 171–393), containing the T-box domain, were PCR amplified from clone RE27439 using 5′ GGGGCCGGATCCCATATGGCACCCAAAATTGTCGGCTCCTGCAAT and 5′ GGGGCCCTCGAGCATCGGATCGCGATCGAAGTCGGTGAGGCG primers. The PCR product was digested with Nde I and Xho I and ligated to a pET-21a vector digested with the same enzymes, resulting in a C-terminal 6xHis-tagged Mid T-box domain. 25 ml of Lauri-Bertani medium was inoculated with an overnight culture of Rosetta-gami cells (Novagen) transformed with the Mid_Tbx_ in pET-21a, grown to an OD of 0.6 and induced with 0.5 mM IPTG. After 3 hours the cells were harvested, resuspended and lysed in 500 µl of buffer containing 20 mM HEPES pH 7.9, 100 mM KCl, 0.2 mM EDTA, 0.2 mM EGTA, 10% glycerol, 0.5 mM DTT, 10 mM imidazole and Complete EDTA-free protease inhibitor (Roche). The lysate was added to 300 µl of Ni-NTA magnetic agarose beads (Qiagen) with the original buffer removed and rocked on ice for 1 hour. The beads were washed 3 times and eluted in the same buffer as above except the washes and elution buffers contained 20 mM and 250 mM of imidazole respectively.

### Site Selection

Site selection was carried out essentially as described [28] with modifications such that it could be carried out non-radioactively. Oligonucleotide R76: CAGGTCAGTTCAGCGGATCCTGTCG(N26)GAGGCGAATTCAGTGCAACTGCAGC, which consists of a 26 nucleotide random core flanked by primer sequences was rendered double stranded using Taq DNA polymerase and primer F (GCTGCAGTTGCACTGAATTCGCCTC), and was purified using High Pure PCR Cleanup Micro Kit (Roche). A 25 µl reaction containing 0.4 ng of purified, double-stranded primer F, 550 ng of purified 6x-His Mid_Tbx_ protein and binding buffer (20 mM HEPES pH 7.9, 100 mM KCl, 0.2 mM EDTA, 0.2 mM EGTA, 20% glycerol, 0.1% Nonidet P40, 0.5 mM DTT, 10 mg/ml BSA, 8 ng/µl poly(dI-dC)•poly(dI-dC)) was assembled and incubated at room temperature for 1 hr. The reaction was added to 10 µl of 5% Ni-NTA magnetic beads (Qiagen) which were washed with binding buffer prior to adding the reaction. The beads were allowed to bind the nucleoprotein complex for 1 hr then washed with 400 µl of wash buffer (20 mM HEPES pH 7.9, 100 mM KCl, 0.2 mM EDTA, 0.2 mM EGTA, 20% glycerol, 0.1% nonidet P40, 0.5 mM DTT, 20 mM imidazole) for 5 min. Following the wash, the bound nucleoprotein complexes were eluted with elution buffer (wash buffer with 250 mM imidazole). 10 µl of the purified complex was PCR amplified with primer F and primer R (CAGGTCAGTTCAGCGGATCCTGTCG) for 15 cycles. The amplification product was purified using High Pure PCR Cleanup Micro Kit (Roche) and quantified using Picogreen (Invitrogen). 0.2 ng of the purified oligonucleotide was used in subsequent rounds of site selection. After 4 rounds of selection, the PCR amplified oligonucleotides were ethanol precipitated and cloned into pCRII-TOPO or pCR2.1-TOPO using TOPO-TA cloning (Invitrogen). Each pCRII or pCR2.1 clone was then sequenced using M13-reverse or M13-forward primers respectively. In total, 54 clones generated usable sequences.

### Motif Generation

The 27 clones which tested positive for binding to Mid in an EMSA were entered into MEME to generate a motif [10]. The relevant parameters were set such that every sequence was used once to generate a motif with a length of 6–26 nucleotides. The sequences of primer F and primer R were used as negative sequences. Motifs in.

Figure 1B were generated using WebLogo [41]. Sequences for the Tbx20 motif were obtained from MacIndoe et al. [6], while those for Mid were generated from data from Liu et al. [18].

### Non-Radioactive Electro-mobility Shift Assays

Probes used for EMSAs were generated from pCRII or pCR2.1 clones by PCR amplification of the cloned oligonucleotide using 5′-biotin-labelled primer F and primer R (see above for sequence). The PCR product was phenol/chloroform extracted and ethanol precipitated in the presence of glycogen. The T-site probe corresponding to the Bs.p palindrome (AATTTCACACCTAGGTGTGAAATT) was obtained as a self-complimentary primer with 5′ biotin labels. Tbx20.MacIndoe (GGAGGTGTGAGGCGA and TCGCCTCACACCTCC), mid.Liu (GGAAGTAGGTCAAG and CTTGACCTACTTCC), mid.Najand (CAAGGTGTCAAGGCG and CGCCTTGACACCTTG), mid.Najand Region 1 (CACCCCCCCAAGGCG and CGCCTTGGGGGGGTG) and mid.Najand Region 2 (CAAGGTGTCAAGGAA and TTCCTTGACACCTTG) were ordered as 5′ biotin-labelled primers and annealed to their complement in 1X Taq polymerase buffer. A 10 µl reaction containing 15 fmol of each biotin-labeled probe, 375 ng of purified 6x-His MidTbx and binding buffer (20 mM HEPES pH 7.9, 100 mM KCl, 0.2 mM EDTA, 0.2 mM EGTA, 20% glycerol, 0.1% nonidet P40, 0.5 mM DTT, 10 mg/ml BSA, 8 ng/µl poly(dI-dC)•poly(dI-dC)) was assembled and incubated at room temperature for 1 hr. The sample was loaded onto a 8×10 cm 5% polyacrylamide, 2.5% glycerol gel in 0.5X TAE running buffer, pre-run at 85V for 1 hour. mid.Najand oligonucleotides were run on a 10% polyacrylamide gel containing 10% glycerol. Once loaded the sample was run for 5 min at 120 V and 1 hour at 85 V for 5% gels, and 2 hours for 10% gels. The oligonucleotide was transferred onto a Hybond-N+ nylon membrane (Amersham) at 85 V for 30 min in 0.5X TAE. Following transfer, the oligonucleotides were cross-linked to the membrane using a transilluminator and visualized using the chemiluminescent nucleic acid detection module (Pierce) according to the manufacturer’s directions. All probes were run a minimum of 2 times to confirm that Mid_Tbx_ is able to bind and retard their mobility. Probes that showed no binding were run 3–5 times to ensure a negative result.
